# Practical Notes on Diagnosis and Treatment

**Published:** 1907-06-08

**Authors:** 


### Practical Notes on Diagnosis and Treatment.

### Treatment of Boils.

One part of chloral hydrate in six parts of cherry laurel water is recommended as a local application to boils. It is applied on spongio-piline.

### Spasmodic Asthma.

To stop the attack a teaspoonful of the following may be given every half-hour until the patient is relieved:—Chloroform ʒj, ether ℥iss, syrup of acacia ʒj, compound tincture of cardomoms ℥j.

### Eczema Intertrigo.

Salol ʒss mixed with powdered starch ʒiiss has proved serviceable and curative in cases of eczema intertrigo. It gives prompt relief to the local irritation.

### Acute Tonsillitis.

Pure guaiacol applied locally by means of a cotton swab is said promptly to check tonsillitis. In most cases two applications are sufficient. Care must be taken that none of the drug reaches the larynx.

### Injection of Cocaine.

Cocaine should be injected into the substance of the skin or mucous membrane, and not into the submucous or subcutaneous tissue. The injection, that is, should be intradermic, and not hypodermic. In this way the risk of introduction into a vein is avoided.

### Sub-acute Cystitis in Women.

In the very large majority of women it is quite impossible for the subject completely to empty the bladder while lying perfectly flat on the back. Therefore, if women, on account of long illness, are placed flat on the back sufficiently long, some urine is almost certainly retained and in consequence changes are prone to take place in the urine which give rise to symptoms of cystitis. In such a case the treatment should not be by washing out and the administration of drugs, but by placing the trunk as soon as possible in a position in which the bladder can be emptied.—*Sir Wm. H. Bennett.*

### Extract of Cotton Seeds in Deficient Lactation.

Professor Gilbert has tested the effect of an extract of cotton-seeds given to nursing mothers in whom the supply of breast milk was deficient. He has obtained results which encourage the hope that the extract will enable nursing to be continued in such cases.

### Paraganglin in Prolapse of the Rectum.

Paraganglin, an extract of the medullary substance of the supra-renal gland, is recommended in prolapse of the rectum in children. It is usually applied locally, but has also been given successfully by the mouth. Dose: 3 to 40 minims in twenty-four hours for an infant of one year.—*British Medical Journal.*

### Asthma in Childhood.

A good many cases improve with advancing age, and the affection may cease at or before puberty. Emetics are of great service in the acuter attacks, and lobelia and belladonna have much remedial value in the less severe. Of all the inhalations I have used the fumes of nitre seem to me the best.—*Dr. H. B. Donkin.*

### Opium in Graves's Disease.

Belladonna and digitalis are no doubt useful and have their place in the treatment of this disease; but opium in full doses and at regular intervals not only soothes the nervous distress and palpitation, but arrests the diarrhœa and vomiting, which all other drugs seem powerless to control. It is in these complications that the chief danger lies.—*Dr. W. B. Cheadle.*

### Carbolic Acid Hypodermically.

To relieve such painful conditions as muscular rheumatism, neuralgia, sciatica, &c., Dr. André Martin has recommended the hypodermic use of carbolic acid: He uses 10 to 15 minims of the following solution: Pure crystallised carbolic acid 2 parts, alcohol or neutral glycerine 2 parts, distilled water 100 parts. One to three injections may be given daily.

